# Characterization and comparison of flavor compounds in different specialty chicken meat after stewing

**DOI:** 10.1016/j.fochx.2025.102589

**Published:** 2025-05-27

**Authors:** Wenqian Lei, Zhaowei Cui, Wanqi Hu, Mengxuan Wang, Xuhua Chen, Xiaojia Hu, Prince Chisoro, Dong Han, Jianchuan Zhou, Chunhui Zhang

**Affiliations:** a Laboratory of Agro-Products Processing, Ministry of Agriculture and Rural Affairs, Institute of Food Science and Technology*,* Chinese Academy of Agricultural Sciences*,* Beijing *100193,* China; b Techlex Food Co.Ltd., Feiyun Avenue, High-tech District, Mianyang City, Sichuan 621006, China

**Keywords:** Specialty chicken meat, Stewing, Taste compounds, Volatile compounds

## Abstract

To explore the flavor changes in specialty chicken meat after stewing, gas chromatography-olfactometry mass spectrometry (GC-O-MS) and gas chromatography ion mobility spectroscopy (GC-IMS) were used to analyze the flavor compound differences among Yellow feather chicken (YC), Lohmann powder chicken (LC), and Jianmen native chicken (JC) after stewing. Results indicated that LC had high shear force and hardness, while JC had lower protein content than YC. Regarding taste compounds, LC had higher contents of umami amino acids and AMP, whereas JC had more sweet amino acids. 13 key aroma compounds were identified by GC-O-MS, like hexanal gives JC a grassy flavor, while dimethyl disulfide affecting only LC's overall flavor. The results were further visualized using GC-IMS, indicating that the total concentration of volatile compounds in LC and JC was higher than in YC. Overall, LC and JC had excellent flavor performance than YC after stewing, with LC showing particularly prominent umami.

## Highlights

• 13 volatile compounds are considered to be odor-active compounds.
• Hexanal and nonanal imparted more grassy and fatty flavor to JC, while dimethyl disulfide contributed only to the overall flavor of LC.
• The umami of LC is particularly prominent, mainly in the high content of Glu and AMP.
• LC and JC showed superior flavor profiles compared to YC after stewing, indicating differences in their flavor compounds.

## 1 Introduction

The consumption of chicken meat has increased globally due to its role as one of the important sources of animal protein (Katemala et al., 2022). In particular, specialty chicken meat, with its rich nutrients, chewy texture and unique flavor, is important in meeting consumer demand for high quality food. Jaturasitha et al. (2017) found that Thai native chickens have smaller muscle fiber diameters, lower fat and cholesterol levels, offering a unique taste. Devatkal et al. (2018) noted that native chickens have higher a* values and water-holding capacity, with significantly lower fat content (*P < 0.05*) than commercial chickens. Moreover, their meat is firmer and richer in odor-active compounds, making them popular among consumers. Additionally, Yang et al. (2018) research shows that all four specialty chicken breeds are characterized by high protein, low fat, and a rich content of essential fatty acids, meeting the nutritional needs of consumers for meat products. Therefore, as consumers' preference for high-quality food continues to grow, a more in-depth and systematic study into the nutritional components and flavor compound composition in specialty chicken becomes particularly important.

Specialty chicken meat can be cooked in a variety of ways, such as stewing, boiling, stir-frying, roasting, etc. In China, boiled chicken is a common method, which not only significantly improves its texture and mouthfeel but also imparts a distinctive flavor to the meat. During the stewing process, cross-linking of myofibrillar proteins in chicken meat accompanied by contraction of muscle segments, while collagen was gradually dissolved at high temperatures (Xu et al., 2020), which led to a softer and tender organization of the chicken meat, further enhancing its edible quality. In addition, the content of taste substances increased accordingly. Wang et al. (2018) explored the effects of two cooking methods on the flavor components of yellow feather chicken. The results showed that the content of organic acids and 5′-nucleotides in stewed samples was higher than that of roasting. Besides, during this process, thermally unstable polyunsaturated fatty acids (PUFA) are susceptible to oxidative degradation, generating a series of volatile compounds (Xu & Yin, 2024), which are the main flavor components in stewed chicken products and play a crucial role in the overall flavor of the finished products. In addition, Yao et al. (2022) studied volatile compounds changes in Dezhou braised chicken during processing. A total of 37 volatiles were identified during processing, and the stewing process was found to be an important stage for flavor impartation of herbs and spices. Currently, the above studies regarding chicken meat have mainly focused on eating quality and flavor compounds during processing. However, the available information on cooked special chicken meat by thermal processing was limited, and there is a lack of systematic studies on the impact of special chicken meat from different breeds after stewing on flavor characteristics.

Therefore, in order to investigate the changing law of flavor characteristics of the specialty chicken meat after stewing, a control group (YC) and a treatment group (LC and JC) were selected as experimental subjects. The changes in color, protein, fat, amino acid and nucleic acid of chicken meat after stewing were determined, and the volatile flavor compounds after stewing were identified by gas chromatography-ion mobility spectrometry (GC-IMS) combined with gas chromatography-olfactometry mass spectrometry (GC-O-MS), which provided a theoretical basis for the formation of the quality of the different specialty chicken meat in the stewing processing.

## 2 Materials and methods

### 2.1 Sample treatment

Jianmen native chickens (JC, n= 6, aged 10–12 months, 1.8–2.0 kg of carcass weight), Lohmann powder chickens (LC, n = 6, aged 10–12 months, 1.6–1.8 kg of carcass weight), and Yellow feather chickens (YC, n = 6, aged 10–12 months, 1.8–2.0 kg of carcass weight) were provided by Sichuan Tieqilishi Group Co., Ltd. All breeds of chickens were fed with the same diet under the same conditions and had similar genetic backgrounds. After slaughter (strictly following the “GB/T 19478–2018” standard), the chickens were vacuum-packed and transported to the laboratory by cold chain transportation at 0–4 °C for subsequent treatment.

The leg and breast muscles of chicken were split, the skin, connective tissues and visible fat were removed from the muscles (The basic composition of the raw chicken meat is shown in Table S1). The 12 muscles of each breed were thoroughly mixed and then randomly divided into two groups (six in each group). Subsequently, they were placed in a polyethylene plastic bag, and a certain amount of water was added according to the solid-liquid ratio of 1: 1.5. Then they were boiled at 100 °C for 30 min, removed and then cooled to room temperature. After cooking, one group was randomly selected for the determination of texture, shear force, electronic nose, electronic tongue and volatile compounds. The remaining one group was blended in a blender (TXTD481, SU-POR, China) for the other essential components. All samples were finally stored in an ultra-low temperature refrigerator at −80 °C for backup, and the experiment was repeated twice to ensure the stability of the samples and the accuracy of the experimental data.

### 2.2 physicochemical quality

#### 2.2.1 Color analysis

The color of chicken samples was determined using a colorimeter (CR-400 Konica Minolta, Japan). Firstly, a white calibration plate was used for calibration, and then different areas of each chicken sample were measured three times and averaged, and the results were expressed as luminance value (L*), redness value (a*), and yellowness value (b*). The total color difference value (*ΔE*) was calculated using the formula (1):

(1) $$∆E=\sqrt{{\left(∆L^{*}\right)}^{2}+{\left(∆a^{*}\right)}^{2}+{\left(∆b^{*}\right)}^{2}}$$

ΔL*, Δa* and Δb* were the difference between the luminance value, redness value and yellowness value of the sample and the standard sample, respectively.

#### 2.2.2 Determination of protein content, fat content and cooking loss

The protein content, fat content and cooking loss of chicken samples were determined. The protein content was measured using the Kjeldahl analysis method in the national standard for food safety (GB 5009.5–2016), and the fat content of the samples was detected using the Soxhlet method in the national standard (GB 5009.6–2016). The cooking loss determination for breast and leg muscles of chicken involves cutting the meat sample into small pieces of approximately 2 cm × 2 cm × 3 cm and weighed as m_1_. The samples were placed in a cooking bag and heated in a water bath at 100 °C for 30 min, then the samples were taken out, cooled to room temperature under running water, dried, and then weighed again, and counted as m_2_. The cooking loss was calculated by the formula (m_1_-m_2_) /m_1_ × 100 %.

#### 2.2.3 Measurement of shear and texture

The samples were divided into columns of 2 cm × 1 cm × 1 cm, with seven samples each. A tenderness meter was used to shear the sample columns along the direction of vertical fibers and the results were recorded. The texture measurement of chicken samples. The breast and leg muscles of chicken were cut into 1 cm × 1 cm × 1 cm squares along the direction of the muscle fibers and determined using a tenderizer (TMS-PRO, FTC, USA). Probe (P35). Measurement parameters were set as follows: test speed 120 mm/min, trigger force 2 N, deformational variable 40 %, and interval time 5 s.

### 2.3 Non-volatile substances determination

#### 2.3.1 Analysis of free amino acids

The determination of free amino acids in meat samples was based on the method of Geng et al. (2018) with slight modifications. 5 g of the meat treatment samples was weighed accurately. After addition of add 20 mL of ultrapure water, homogenize 3 times at 18000 r/min (10 s each time, 10 s interval). Afterwards, 20 mL of 5 % (V/V) trichloroacetic acid solution was added mixed and then the mix was left in the refrigerator at 4 °C for 12 h. Filter with qualitative filter paper, and the filtrate was first adjusted to pH 6.0 with 4 mol/L KOH, and then fixed with ultrapure water to 50 mL. 1 mL of the filtrate was filtered through a 0.45 μm membrane. Pipette 10 μL of the filtrate, add 70 μL of AccQ-Fluor Buffer to the derivatization tube, and then pipette 20 μL of ready-made AccQ-Fluor derivative into the derivatization tube, keep vortexing and mixing for 10 s. After incubating for 1 min, and heat it up in an oven at 55 °C for 10 min, and then take it out for HPLC detection. The chromatographic column was Nova-PakTM C18 column (150 mm × 3.9 mm); column temperature: 37 °C; UV detection wavelength: 248 nm; injection volume: 10 μL; flow rate: 1 mL/min; mobile phase A: diluted with ultrapure water according to 1:10 (V/V); mobile phase D: acetonitrile (chromatographically pure); mobile phase C: ultrapure water.

#### 2.3.2 Nucleotide determination

The method of Kaewsatuan et al. (2023) was referred and slightly modified. The preparation of standards were set as follows, 100 mg of adenosine monophosphate (AMP), guanosine monophosphate (GMP) and inosine monophosphate (IMP) were weighed, dissolved in 100 mL volumetric flasks with ultrapure water and fixed in volume to make a standard solution with a concentration of 1.0 mg/mL. Afterwards, 25, 50, 100, 250, 500, 750, 1000 μL of each standard solution were taken into a 10 mL volumetric flask, and then dilute it with ultrapure water to 10 mL. After shaking the mix, the solution was filtered through a 0.45 μm microporous filter membrane. For the sample treatments: Take 5 g meat sample in a 50 mL centrifuge tube, add 15 mL of 5 % perchloric acid solution at 4 °C, homogenize 3 times at 18000 r/min (10 s each time, with an interval of 10 s) and then centrifuged at 4000 r/min for 5 min, take the supernatant and transfer it to a 100 mL beaker, and the residue was oscillated with 10 mL 5 % perchloric acid for 5 min, centrifuged, and the supernatant was combined. The pH was adjusted to 6.5 with 1 mol/L NaOH, and the solution was shaken with 50 mL of ultrapure water and passed through a 0.45 μm filter membrane for use. HPLC conditions: chromatographic column Intersil ODS-3 (250 mm × 4.6 mm); UV detector; detection wavelength: 260 nm; column temperature: 30 °C; mobile phase A was methanol, and B was 0.02 mol/L potassium dihydrogen phosphate solution; the mobile phase elution program was 5 % A, 95 % B, elution for 15 min; flow rate: 1 mL/min; injection volume: 10 μL.

#### 2.3.3 Calculation of equivalent umami concentration (EUC)

EUC is the concentration of MSG that is equivalent to the effect of free amino acids combined with 5′-nucleotides. The formula for calculating EUC (g MSG/100 g) is as follows (Han et al., 2021):

(2) $$\begin{array}{c} \mathrm{EUC}=\sum \alpha_{i}\beta_{i}+1218\left(\sum \alpha_{i}\beta_{i}\right)\left(\sum \alpha_{j}\beta_{j}\right) \end{array}$$

α_i_ is the concentration of the umami amino acids (Asp or Glu), β_i_ is the relative umami flavor concentration (RUC) of Glu and Asp with respect to MSG, which are 1 and 0.077, respectively. α_j_ is the concentration of each umami 5′-nucleotide (AMP, GMP, IMP), β_j_ is the concentration of the IMP (AMP, 0.18; GMP, 2.3) the RUC of each umami 5′-nucleotide. The concentrations used in the calculations are g/100 g and 1218 is the synergistic constant.

#### 2.3.4 Electronic tongue

A 3 g of sample was weighed into a 50 mL centrifuge tube, then 20 mL of deionized water was added, homogenized, and ultrasonicated for 15 min, followed by centrifugation at 1000 r/min for 20 min, and the supernatant was withdrawn and filtered through a funnel, and the filtrate was fixed to 100 mL and homogenized by transferring it to specific sample cups for the determination of the electronic tongue (ASTREE, AlphaMOS, Toulouse, France). Before the determination, the sensors were calibrated and the electrodes were activated for 24 h using a reference solution (30 mM KCl + 0.3 mM tartaric acid). During the test, the sensor was cleaned in the cleaning solution and the reference solution and subsequently immersed in the sample solution for 60 s for detection.

### 2.4 Volatile compound profile analysis

#### 2.4.1 Electronic nose

Meat samples were measured using an electronic nose (PEN 3, Airsense, GER, all sensors and their applications are shown in Table S2). The samples were chopped and accurately weighed within 2 min to 2.00 g in a 20 mL headspace vial, which was then immediately sealed the vial with a headspace cap that has a PTFE silicone septum. All samples were equilibrated at room temperature for 30 min, with a test time of 60 s and a wash time of 180 s for each sample.

#### 2.4.2 GC-O-MS analysis

Using the method Yan et al. (2024), was some slightly modifications. The volatile compounds in chicken samples were determined using GC-O-MS (GC-O-MS QP2010, Shimadzu Corporation, Japan). A 4 g sample was accurately weighed in a 50 mL headspace vial within 2 min, and then 1 μL of 0.408 μg/μL 2-methyl-3-heptanone (solvent methanol) was added as an internal standard. After completion of the addition, the headspace vial was immediately sealed using a cap with a PTFE silica gel gasket. The headspace vial was equilibrated in a water bath at 60 °C for 30 min, and then the volatile compounds in the headspace vial were extracted using a 50/30 μm DVB/CAR/PDMS fiber tip. After 30 min of extraction, the head was immediately transferred to the injection port at 240 °C for desorption for 5 min. GC conditions: the column was DB-WAX (30 m × 0.18 mm × 0.18 μm) with a split ratio of 20:1; ramp-up procedure: injection temperature was 240 °C, initial column temperature was 40 °C, held for 5 min, ramped up to 100 °C at 5 °C/min, then ramped up to 120 °C at 3 °C/min, and finally to 230 °C at 5 °C/min, and held for 5 min; MS conditions: ion source temperature 200 °C, ion source energy 70 eV. The full-scan mode was used, and the mass scanning range was 35–500 *m*/*z*.

Qualitative analysis: The mass spectrometry results were compared with the National Institute of Standards and Technology (NIST) database to identify volatile compounds in the samples by comparing retention indices (RI). The volatile flavor compounds detected all had a match greater than 80, with a maximum match of 100. The formula for calculating the retention index is as follows:

(3) $$\begin{array}{c} \mathrm{RI}=\frac{t_{i}-t_{n}}{t_{n+1}-t_{n}}\times 100+n\times 100 \end{array}$$

where t_i_, t_n_ and t_n+1_ are the retention times (min) of the sample, n-alkanes C_n_, and n-alkanes C_n+1_, respectively.

Semi-quantitative analysis: The internal standard method was used. The concentration and peak area of the internal standard (2-methyl-3-heptanone) were used to calculate the relative concentration of volatile compounds to be measured.

The sniff test of the GC-O system was carried out by three professionally trained sensory assessors (2 males and 1 female) trained in GB / T 16291.1–2012. Throughout the assessment process, the assessor was required to record the retention time, intensity and character of the odor as soon as it was perceived. Each sample was evaluated 3 times, with one evaluator performing the sensory each time. An odor-active compound is considered to be a valid odor-active compound if it is detected in at least 2 of the 3 GC-O analyses (detection frequency (DF) ≥ 2).

#### 2.4.3 Calculation of relative odor activity values (ROAVs)

The ROAVs is the ratio of the relative concentration of a volatile compound to its threshold value (Yan et al., 2024). When ROAV >1, it means that the volatile compound contributes to the overall flavor.

#### 2.4.4 GC-IMS analysis

Volatile compounds in chicken samples were determined by gas chromatography-ion mobility spectrometry (Flavor Spec®, Gesellschaft für Analytische Sensorsysteme mbH, Germany) under the conditions described in the available literature (Li et al., 2024) with minor modifications. The sample was weighed into a 20 mL headspace vial (the procedure was repeated to prepare three parallel samples) and incubated at 60 °C for 15 min with shaking and heating at a speed of 500 r·min^−1^. The temperature of the injection needle was set at 70 °C, and 500 μL of the sample was injected into the injection port of the gas chromatograph (GC) through the automated headspace injection system for analysis. The analysis was performed on a FS-SE-54-CB-1 column (15 m × 0.53 mm, 1 μm) at a column temperature of 60 °C for 24 min. The carrier gas was nitrogen (≥ 99.999 %), and the flow rate was 2 mL·min^−1^, held for 2 min, then increased to 20 mL·min^−1^ for 2–8 min, 100 mL·min^−1^ for 8–18 min, and finally increased to 150 mL·min^−1^ for 18–23 min. The GC-IMS data were obtained using Laboratory Analytical Viewer (LAV) software, and the volatile compounds in the samples were identified by combining the NIST2014 and IMS databases integrated in the GC-IMS Library Search software. In addition, the Reporter plug-in was utilized to compare the spectral differences between the samples, and the Gallery Plot plug-in was used to create fingerprints of the volatile compounds in the samples.

### 2.5 Data analysis

Analysis of variance (ANOVA) was performed using R software (ver. 4.1.2), followed by analysis of significance of differences using Duncan’ s Multiple Test, which was considered statistically significant when *P < 0.05*. All graphs were produced by Graphpad Prism (ver. 9.5.0) software, origin software (ver. 2024), R software (ver. 4.1.2) and online website (https://www.bioinformatics.com.cn/).

## 3 Results and discussion

### 3.1 Basic composition analysis and edible quality

#### 3.1.1 Protein content, fat content and cooking loss

The protein content, fat content, and cooking loss results of YC, LC and JC are shown in Table 1. Breeds had a significant effect (*P < 0.01*) on protein content, with JC had the lowest protein content in leg and breast muscles with 32.31 g/100 g and 32.89 g/100 g, respectively. The content of intramuscular fat directly affected the juiciness and tenderness of meat (Yu et al., 2023). The results showed that in leg muscle, the fat content was significantly lower (*P < 0.05*) in LC and JC than in YC, inferring that the juiciness and texture of YC may be better in leg muscle than in LC and JC. In breast muscle, JC had the significantly lowest fat content (*P < 0.05*) of 0.92 g/100 g. The results on cooking loss showed no significant differences (*P > 0.05*) in chicken breast muscle from different breeds. However, among the leg muscle, the cooking loss of LC was significantly lower (*P < 0.05*) than that of YC, which could be attributed to the high internal energy expenditure of LC during laying, resulting in firmer meat with lower water content.

**Table 1:** Protein content, fat content and cooking loss of two muscles in different chicken breeds.

Index	Breed (B) and Muscle (M)						Significance (*P* value)		
	Leg muscle			Breast muscle					
	YC	LC	JC	YC	LC	JC	B	M	B × M
Protein (g/100 g)	33.73 ± 0.28^ABb^	35.26 ± 1.02^Aa^	32.31 ± 1.57^Ba^	36.69 ± 0.91^Aa^	35.34 ± 1.59^Aa^	32.89 ± 1.03^Ba^	**	*	NS
Fat (g/100 g)	4.49 ± 0.18^Aa^	2.78 ± 0.44^Ba^	2.50 ± 0.51^Ba^	2.47 ± 0.58^Ab^	2.17 ± 0.19^Aa^	0.92 ± 0.41^Bb^	***	***	*
Cooking loss (%)	40.30 ± 1.66^Aa^	36.45 ± 0.79^Ba^	38.64 ± 1.79^ABa^	32.59 ± 1.27^Ab^	32.47 ± 2.17^Aa^	33.45 ± 2.75^Ab^	NS	***	NS

Note: Results were expressed as mean ± standard derivation. YC, LC and JC refers to Yellow feather chicken, Lohmann powder chicken and Jianmen native chicken, respectively. Different lowercase letters (a-b) indicate a significant difference (*P < 0.05*, differences between muscle) within the same breed. Different capital letters (A-B) indicate a significant difference (*P < 0.05*, differences between breed) within the same muscle type. Significance: *** *P < 0.001*, ** *P < 0.01*, * *P < 0.05*; NS, not significant.

#### 3.1.2 Color analysis

**Fig. 1:**
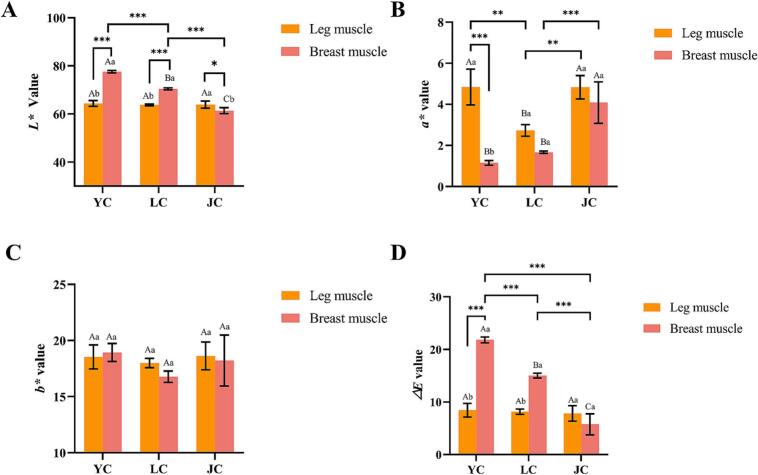
The color of leg muscle and breast muscle in Yellow feather chicken (YC), Lohmann powder chicken (LC) and Jianmen native chicken (JC). L* (A), a* (B), b* (C) and *△E* (D) were measured. Different lowercase letters (a-b) indicate a significant difference (*P < 0.05*) within the same breed. Different capital letters (A-C) indicate a significant difference (*P < 0.05*) within the same muscle type. Significance: *** *P < 0.001*, ** *P < 0.01*, * *P < 0.05*.

Meat color is considered to be an important factor influencing consumers' purchasing decisions, which is mainly dependent on the myoglobin in the muscle (Suman & Joseph, 2013). As can be seen from Fig. 1, The L>* value and a>* value were the main reasons for the color differences. There was a significant difference in L* between YC, LC and JC in the breast muscle (*P < 0.05*), with the lowest value of L* of JC, 61.37. For redness value (a*), LC had significantly lower a* values (*P < 0.05*) in leg muscle than YC and JC, which could be attributed to the higher levels of lipid oxidation and free radicals production occurring in their muscles (Arshad et al., 2017), leading to a lower a* value, which is in agreement with the findings of Cho et al. (2015). In addition, the a* value of leg muscle was higher than that of breast muscle, due to the fact that leg muscle consists mainly of red muscle fibers, which contain 5–10 times more myoglobin than chicken breast muscle, making the meat redder. The values of total color difference (*∆E*) of the three chicken breeds were all more than 1, which indicates that the color differences were sufficiently easy to be recognized by the consumer (Altmann et al., 2022).

#### 3.1.3 Texture and shear force

Table 2 demonstrates the shear force and texture of YC, LC, and JC in leg and breast muscles. The results showed that shear force, hardness, and chewiness were significantly (*P < 0.05*) affected by breeds and muscle types. Shear force reflected the tenderness of the muscle. Lower shear force indicates higher intermuscular water content and greater tenderness. The leg and breast muscles of LC had the significantly higher shear force (*P < 0.05*) of 60.50 and 42.10 N, respectively. This may be attributed to the thickening of myofibrils in LC as the age of the chickens increases, cross-linking of connective tissue and a decrease in collagen solubility (Listrat et al., 2016), which thereby affecting meat tenderness. Shear forces varied by muscle types. The shear force of leg muscle was significantly higher (*P < 0.05*) than that of breast muscle, which may be related to the high movement of the leg muscle. In addition, the hardness of LC was significantly (*P < 0.05*) higher than that of YC and JC, which corresponded to the results of the shear force. Chewiness of the leg muscle of YC was significantly higher than that of the other breeds (*P < 0.05*) and chewiness of breast muscle showed less difference among breeds.

**Table 2:** Texture and shear force of two muscles in YC, LC and JC.

Index	Breed (B) and Muscle (M)						Significance (*P* value)		
	Leg muscle			Breast muscle					
	YC	LC	JC	YC	LC	JC	B	M	B × M
Shear force (N)	45.52 ± 6.22^Ba^	60.50 ± 10.96^Aa^	19.61 ± 4.32^Ca^	26.92 ± 3.71^Bb^	42.10 ± 1.54^Ab^	24.90 ± 1.49^Ba^	*	*	*
Hardness (N)	21.02 ± 1.97^Ba^	56.21 ± 8.26^Aa^	21.69 ± 6.63^Ba^	15.97 ± 6.24^Ba^	36.07 ± 7.00^Ab^	17.38 ± 3.06^Ba^	***	**	NS
Cohesiveness	0.50 ± 0.00^Aa^	0.43 ± 0.05^Aa^	0.50 ± 0.00^Aa^	0.50 ± 0.00^Aa^	0.43 ± 0.15^Aa^	0.43 ± 0.06^Aa^	NS	NS	NS
Springiness	2.17 ± 0.11^Aa^	1.46 ± 0.24^Aa^	2.09 ± 0.21^Aa^	2.20 ± 0.18^Aa^	1.96 ± 0.53^Aa^	2.05 ± 0.42^Aa^	NS	NS	NS
Chewiness (N)	17.31 ± 3.42^Aa^	4.33 ± 0.87^Ba^	5.29 ± 2.02^Ba^	9.70 ± 3.09^Ab^	3.91 ± 3.25^Aa^	3.89 ± 1.33^Aa^	***	**	NS

Note: Results were expressed as mean ± standard derivation. YC, LC and JC refers to Yellow feather chicken, Lohmann powder chicken and Jianmen native chicken, respectively. Different lowercase letters (a-b) indicate a significant difference (*P < 0.05*, differences between muscle) within the same breed. Different capital letters (A-B) indicate a significant difference (*P < 0.05*, differences between breed) within the same muscle type. Significance: *** *P < 0.001*, ** *P < 0.01*, * *P < 0.05*; NS, not significant.

### 3.2 Taste compounds analysis

#### 3.2.1 Free amino acids

**Fig. 2:**
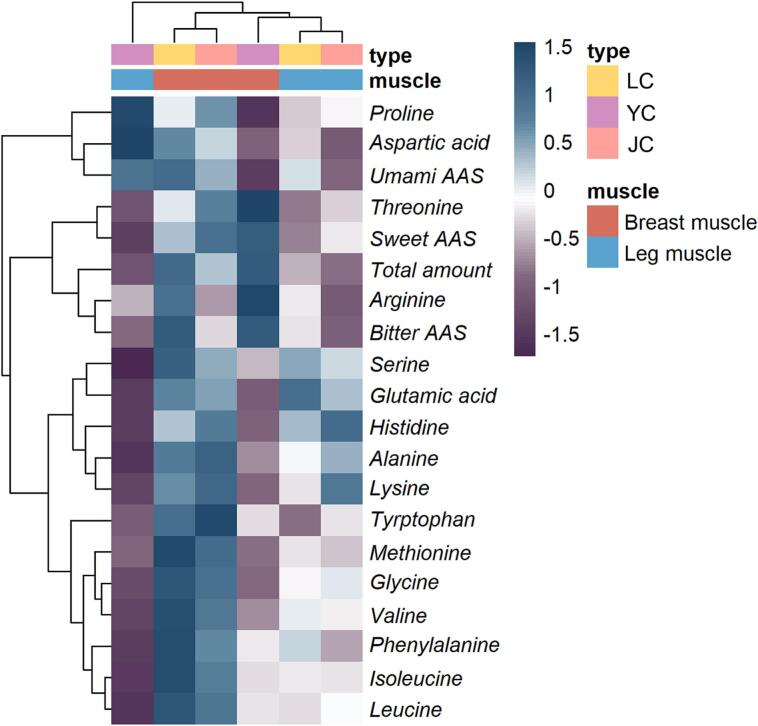
Heatmap clustering of free amino acid of two muscles in YC, LC and JC.

Free amino acids are flavor precursors that can impart tastes like sweetness (Ser, Gly, Thr, Ala, Pro), umami (Asp, Glu), and bitterness (His, Arg, Tyr, Val, Met, Ile, Leu, Phe, Lys) (Ramalingam et al., 2019). The results, as shown in Fig. 2, revealed significant differences (*P < 0.05*) in the total amino acid content of different breeds of chicken meat after stewing. Among these, leg muscle of LC (240.32 mg/100 g) and breast muscle of YC (437.84 mg/100 g) had the highest total amino acid content. Glu, as a umami amino acid, and its content was significantly highest (*P < 0.05*) in LC, which is likely to assess the tastes of LC as well as being an important quality marker for establishing flavor profiles. Sweet and bitter amino acids are the main components in different chicken breeds. Of these, the sweet amino acid can synergize with Glu/Asp to promote umami taste (Wang et al., 2022), and the highest contribution was Thr, which had the highest content of 164.25 mg/100 g in the breast muscle of YC. Phe, Met, Val, Leu and Ile as bitter amino acids had the highest content (*P < 0.05*) in the breast muscle of LC. Whereas, the high content of Met in the breast muscle of LC indicated that its proteins are hydrolyzed during cooking, leading to an increase in amino acid concentration (Yue et al., 2016). In summary, umami amino acids and bitter amino acids collectively have a significant impact on the flavor of the breast muscle of LC, enhancing its unique and rich flavor profile.

Taste activity value (TAV) is commonly used to measure the contribution of a substance to taste. If a substance has a TAV > 1, it means that the substance has an important contribution to the taste (Wang et al., 2020). In Table 3, only Arg had a TAV > 1, which indicates that this amino acid contributes more to the taste of chicken meat.

**Table 3:** TAVs of free amino acid of two muscles in different chicken breeds after stewing.

FAA	Breed (B) and Muscle (M)						Significance (*P* value)		
	Leg muscle			Breast muscle					
	YC	LC	JC	YC	LC	JC	B	M	B × M
Umami FAA									
Asp	0.41^Aa^	0.17^Ba^	0.08^Cb^	0.09^Cb^	0.29^Ab^	0.23^Ba^	***	***	***
Glu	0.36^Cb^	0.84^Aa^	0.71^Ba^	0.45^Ca^	0.78^Ab^	0.72^Ba^	***	NS	***
Sweet FAA									
Ser	0.03^Cb^	0.10^Ab^	0.09^Bb^	0.07^Ca^	0.12^Aa^	0.10^Ba^	***	***	***
Gly	0.02^Ba^	0.05^Ab^	0.05^Ab^	0.03^Ba^	0.08^Aa^	0.07^Aa^	***	***	**
Thr	0.09^Cb^	0.16^Bb^	0.25^Ab^	0.63^Aa^	0.33^Ca^	0.47^Aa^	***	***	***
Ala	0.08^Cb^	0.14^Bb^	0.16^Ab^	0.11^Ba^	0.18^Aa^	0.19^Aa^	***	***	NS
Pro	0.01^Aa^	0.02^Aa^	0.02^Aa^	0.01^Ba^	0.02^ABa^	0.02^Aa^	*	NS	NS
Bitter FAA									
His	0.17^Cb^	0.46^Ba^	0.56^Aa^	0.25^Ca^	0.45^Ba^	0.53^Aa^	***	NS	**
Arg	1.14^Bb^	1.54^Ab^	0.40^Cb^	3.85^Aa^	3.11^Ba^	0.98^Ca^	***	***	***
Tyr	0.02^Bb^	0.03^ABb^	0.05^Ab^	0.04^Ba^	0.08^Aa^	0.08^Aa^	***	***	NS
Val	0.02^Bb^	0.18^Ab^	0.17^Ab^	0.12^Ca^	0.31^Aa^	0.26^Ba^	***	***	NS
Met	0.09^Aa^	0.13^Ab^	0.12^Aa^	0.03^Bb^	0.22^Aa^	0.19^Aa^	***	NS	**
Ile	0.01^Bb^	0.05^Ab^	0.05^Ab^	0.05^Ca^	0.09^Aa^	0.08^Ba^	***	***	NS
Leu	0.01^Cb^	0.04^Bb^	0.04^Ab^	0.04^Ca^	0.08^Aa^	0.07^Ba^	***	***	**
Phe	0.01^Cb^	0.07^Ab^	0.04^Bb^	0.04^Ca^	0.12^Aa^	0.09^Ba^	***	***	NS
Lys	0.01^Cb^	0.04^Bb^	0.10^Ab^	0.17^Ca^	0.19^Ba^	0.21^Aa^	***	***	***

Note: YC, LC and JC refers to Yellow feather chicken, Lohmann powder chicken and Jianmen native chicken, respectively. Different lowercase letters (a-b) indicate a significant difference (*P < 0.05*, differences between muscle) within the same breed. Different capital letters (A-B) indicate a significant difference (*P < 0.05*, differences between breed) within the same muscle type. Significance: *** *P < 0.001*, ** *P < 0.01*, * *P < 0.05*; NS, not significant.

#### 3.2.2 5′-Nucleotides and EUC analysis

**Fig. 3:**
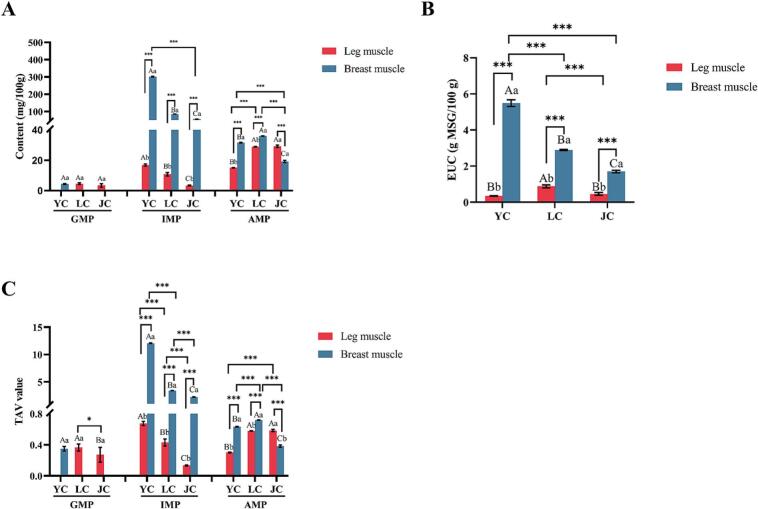
(A) Nucleotides of two muscles in different chicken samples. (B) EUC of two muscles in different chicken breeds. (C) TAVs of three kinds of taste nucleotides. Different lowercase letters (a-b) indicate a significant difference (*P < 0.05*) within the same breed. Different capital letters (A-C) indicate a significant difference (*P < 0.05*) within the same muscle type. Significance: *** *P < 0.001*, * *P < 0.05*.

Nucleotides, such as inosine monophosphate (IMP), guanosine monophosphate (GMP), and adenosine monophosphate (AMP), are important umami compounds in meat products that play a crucial role in regulating meat flavor (Yue et al., 2016). Fig. 3A shows significant differences (*P < 0.05*) in nucleotide content among YC, LC, and JC. Notably, the IMP and GMP of YC was significantly higher than those of LC and JC (*P < 0.05*). This may be because the IMP and GMP in LC and JC are transferred to the chicken broth during stewing or underwent thermal decomposition, resulting in dephosphorylation and deamination reactions that produced inosine and hypoxanthine (Xu & Yin, 2024). According to the taste threshold values of nucleotide, the TAV of IMP is much greater than 1 (Fig. 3C), indicating that this flavoring nucleotide can effectively enhance the umami flavor of chicken meat. Furthermore, although the TAV value of AMP is less than 1, it has a synergistic effect in inducing umami flavor, as it can reduce bitterness and impart a unique sweetness to chicken meat (Dashdorj et al., 2015). The results of the study showed that AMP in breast muscle of LC (36.00 mg/100 g) was significantly higher (*P < 0.05*) than in other breeds, a phenomenon that may be attributed to the conversion of large amounts of energy (ATP) of LC to ADP, AMP under enzymatic activity (Dashdorj et al., 2015). Moreover, dehydration and condensation effect (Skovgaard, 2004) produced during cooking further increased the AMP content. To provide a more intuitive evaluation of the umami characteristics of chicken meat after stewing, the equivalent umami concentration (EUC) was utilized. As shown in Fig. 3B, the EUC range for breast muscle was 1.69–5.49 g MSG/100 g, with higher values observed in YC and LC. In contrast, the ECU range for leg meat was 0.35–0.88 g MSG/100 g, with the highest value found in LC.

#### 3.2.3 *E*-tongue

**Fig. 4:**
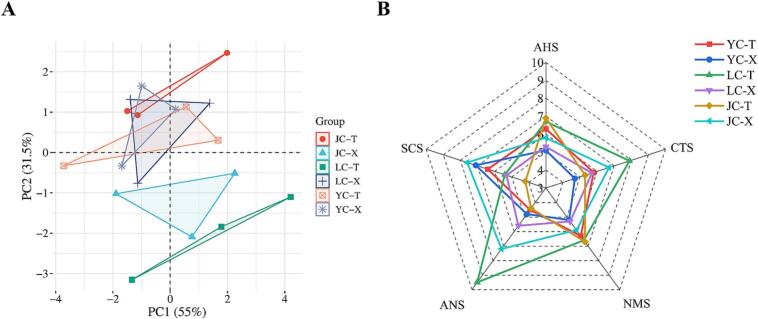
Sensory analysis of two muscles in different chicken samples. (A) Principal component analysis (PCA) score of E-tongue. (B) E-tongue radar chart. YC-T refers to the leg muscle of Yellow feather chicken; YC-X refers to the breast muscle of Yellow feather chicken; LC-T refers to the leg muscle of Lohmann powder chicken; LC-X refers to the breast muscle of Lohmann powder chicken; JC-T refers to the leg muscle of Jianmen native chicken; JC-X refers to the breast muscle of Jianmen native chicken.

Electronic tongue was used to detected the tastes of the two muscles of different breeds of chicken meat. In Fig. 4A, PC1 and PC2 contribute 55.0 % and 31.5 % respectively, with a combined contribution of 86.5 %, which could well reflect all the information of the chicken samples, indicating that the taste performances of the different breeds of chicken meat after stewing had a good discriminatory power. Fig. 4B shows the radargram, where AHS, CTS, NMS, ANS and SCS are sourness, saltiness, umami, sweetness and bitterness sensors, respectively (Zhang et al., 2023). When the flavor precursors of chicken meat are dissolved in saliva, they interact with G Protein-Coupled Receptors (GPCRS) on the tongue (Nishihara et al., 2023) to imparts chicken tastes. The leg and breast muscles of all three chicken breeds responded to the sensors with significant differences in sourness, bitterness, saltiness, sweetness and umami. Of them, the umami and sourness response values of leg muscle were higher than those of breast muscle, especially the high response value of leg muscle of JC and LC. For sweetness and saltiness, the response value of leg muscle of LC was the most prominent, while for bitterness, the response value of leg muscle of JC was the lowest.

### 3.3 Volatile compound analysis

#### 3.3.1 E-nose

**Fig. 5:**
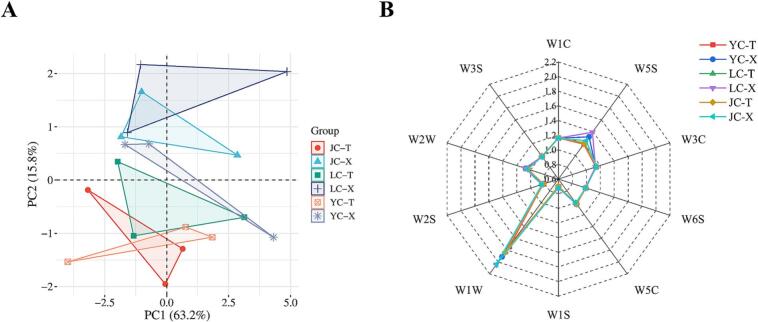
Sensory analysis of two muscles in different chicken samples. (A) Principal component analysis (PCA) score of E-nose. (B) E-nose radar chart.

The electronic nose is an instrument that imitates the biological olfactory system to rapidly detect volatile flavor substances in meat products. Fig. 5A shows that the contribution rates of PC1 and PC2 are 63.2 % and 15.8 %, respectively, totaling 79 %. In breast muscle, the distance between YC and LC indicates significant differences in their aroma characteristics. In contrast, the different chicken breeds in leg muscle are more closely clustered, suggesting minor differences in their aroma components. As shown in Fig. 5B, the volatile components of different breeds of chicken meat showed certain differences, particularly reflected in their sensitivity to sensors W1W and W5S. Among them, the breast muscle of LC and JC showed the highest response values on W1W and W5S. The response values of sensors W3S, W1C, W3C, W6S, W5C, W1S, W2S, and W2W were largely concentrated at a single point, indicating that they corresponded to more similar overall volatile odorants. Overall, there are differences in aroma among different chicken breast muscle, while the aromas of leg muscle are relatively similar.

#### 3.3.2 GC-O-MS results and quantitation of the odor-active compounds

Volatile flavor compounds were determined by GC-O-MS in the leg and breast muscles of YC, LC, and JC after stewing. As shown in Table S4, a total of 41 volatile compounds were identified, with YC, LC, and JC containing 29, 24, and 24 compounds, respectively. The main compounds included aldehydes, alcohols, ketones, acids, esters, ethers, hydrocarbons, and heterocyclics compounds. Table 4 presents the content and proportion of volatile compounds in different chicken breeds. Aldehydes had the largest percentage (25.6 %–82.8 %) with a total content of 372.70–1248.97 μg/kg, followed by alcohols (0.6 %–32.3 %) with a total content of 172.16–591.98 μg/kg. They were closely related to the flavor of the meat, with the largest percentage of aldehydes having the highest content in the leg muscle of JC, while the total alcohols having the highest content in the breast muscle of YC.

**Table 4:** Categories and concentrations of the volatile compounds in two muscles of different chicken samples after stewing.

Classes of components	Concentration μg/kg (Ratio%)					
	Leg muscles			Breast muscles		
	YC	LC	JC	YC	LC	JC
Aldehydes	892.27 (52.6)	602.48 (42.3)	1248.97 (82.8)	606.72 (34.9)	372.70 (25.6)	514.13 (51.8)
Ketones	547.61 (32.3)	221.14 (15.5)	35.11 (2.3)	16.44 (0.9)	8.94 (0.6)	9.41 (0.9)
Alcohols	217.55 (12.8)	574.20 (40.3)	172.16 (11.4)	591.98 (34.1)	453.36 (31.2)	421.49 (42.5)
Acids	2.05 (0.1)	7.97 (0.5)	ND	49.99 (2.9)	12.13 (0.8)	ND
Esters	9.35 (0.5)	2.00 (0.1)	30.25 (2.1)	313.12 (18.1)	ND	9.34 (0.9)
Ethers	ND	ND	ND	127.00 (7.3)	286.89 (19.7)	ND
Heterocyclics	24.59 (1.4)	19.19 (1.3)	21.62 (1.4)	31.84 (1.8)	43.23 (3.0)	39.65 (3.9)
Hydrocarbons	4.47 (0.3)	ND	ND	ND	276.78 (19.1)	ND

Note: YC, LC and JC refers to Yellow feather chicken, Lohmann powder chicken and Jianmen native chicken, respectively. “ND” stand for “Not Available” (The volatile compounds was not detected in this sample).

**Fig. 6:**
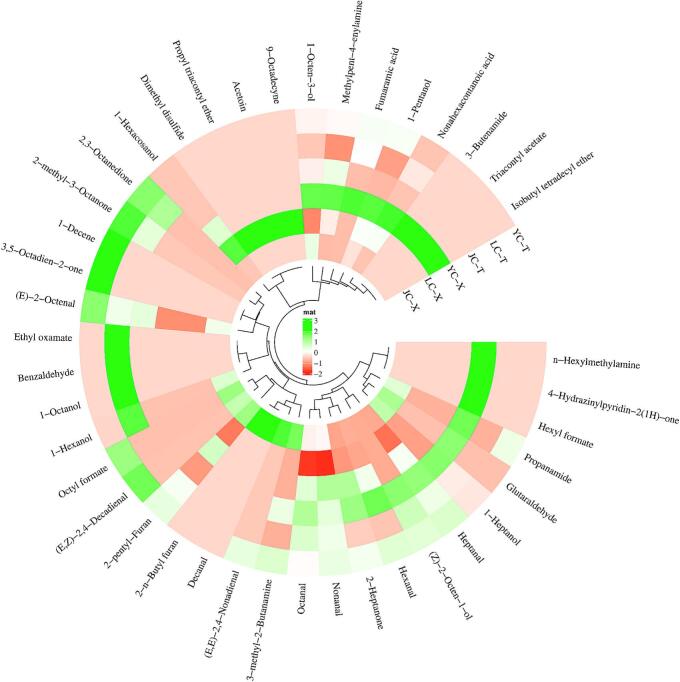
Circular clustering heatmap of volatile compound content in two muscles of different chicken samples after stewing.

Aldehydes are the primary odor compounds during chicken stewing process (Bi et al., 2021). As shown in Fig. 6, a total of 10 aldehydes were detected, including hexanal, heptanal, nonanal, octanal, 2-octenal, benzaldehyde, (*E,Z*)-2,4-decadienal, (*E,E*)-2,4-nonadienal, among others. Hexanal and heptanal are produced from the oxidation of linoleic and arachidonic acids, respectively (Al-Dalali et al., 2022), and their contents were highest in the leg muscle of JC at 1076.40 μg/kg and 34.85 μg/kg, respectively. Nonanal, which is derived from the oxidation of oleic acid (Tanimoto et al., 2015) and was found in the highest amount (104.88 μg/kg) in the breast muscle of YC. Benzaldehyde, which has a nutty flavor, originates from the Strecker degradation of phenylalanine (Luo et al., 2022), contributing to the flavor of chicken, and it was detected only in the leg muscle of LC (21.66 μg/kg). Previous studies have reported that unsaturated fatty acids (USFA) are more abundant in chicken compared to saturated fatty acids (SFA), accounting for nearly half of the total fatty acid content (Soares et al., 2009). The high levels of USFA undergo oxidation during heating, generating significant amounts of volatile unsaturated aldehydes (Yao et al., 2022). (*E,Z*)-2,4-decadiene, a volatile flavor compound widely recognized in cooked chicken meat as an important contributor to chicken meat aroma (Zhang et al., 2018), was detected only in the breast muscle of JC and the leg muscle of YC, which may be related to the oxidation of linoleic acid (Du et al., 2020). Other unsaturated aldehydes, such as (*E,E*)-2,4-nonadienal, were only found in the leg muscle of YC and the breast muscle of JC. This may be closely related to their higher proportions of oleic acid, which leads to the formation of more 2-alkenals during the cooked chicken (Dashmaa et al., 2011).

Alcohols are mainly produced through the oxidative degradation of unsaturated fatty acids (Zhang et al., 2018). During high-temperature stewing, monounsaturated fatty acids (MUFAs) are oxidatively broken down to hydroperoxides, which further decompose into aldehydes (Wu et al., 2021). These aldehydes are then reduced to their corresponding alcohols by aldehyde reductase. A total of 7 alcohols were detected after stewing, among which, 1-octen-3-ol and 1-hexanol were the main alcohols. 1-octen-3-ol was found to be the highest in the breast muscle of YC at 325.41 μg/kg, which may be closely related to its higher proportion of C20:4n6 (Sohail et al., 2022). Additionally, there were significant differences (*p < 0.05*) in the content of 1-octen-3-ol between different muscle type of the same breed. It was shown that the higher content of 1-octen-3-ol in breast muscle than in leg muscle. This disparity arises from significant variations in fatty acid composition. Research indicates that the total fatty acids, n-3 and n-6 PUFAs content in leg muscle are notably higher than in breast muscle (Xiao et al., 2021). 1-Hexanol is derived from the oxidation of linoleic acid to hexanal, followed by partial reduction catalyzed by aldehyde reductase (Zhang et al., 2015), and its content was highest in the leg muscle of LC (459.48 μg/kg).

In total, 5 fatty ketones were detected, including 2-heptanone, 2,3-octanedione, 4-hydrazinylpyridin-2(1H)-one, and (*E,E*)-3,5-octadien-2-one. Notably, 4-hydrazinylpyridin-2(*1H*)-one was only detected in the leg muscle of JC, while (*E,E*)-3,5-octadien-2-one was only found in the leg muscle of YC. Additionally, 9 heterocyclics compounds were identified in the volatile compounds of cooked chicken, primarily including 2-pentylfuran, 2-n-butyl furan, acetoin, and dimethyl disulfide. 2-Pentylfuran and dimethyl disulfide were the key furans and sulfides in cooked chicken meat. In which, 2-pentylfuran was found at a higher concentration in the leg muscle of JC, measuring 42.89 μg/kg, which may be associated with a higher content of C18:2n6c (Sohail et al., 2022). Sulfur-containing compounds have been identified as important contributors to the aromatic characteristics of chicken broth and cooked chicken. Dimethyl disulfide was detected only in the breast muscle of LC at 8.97 μg/kg, which was attributed to the high content of Met in the breast muscle of LC. It is degraded by Strecker degradation to produce methional, which is further decomposed rapidly to methanethiol and finally to dimethyl disulfide and other sulfide compounds (Moon et al., 2006), giving chicken meat its distinctive flavor. In addition, 2 acids, 4 esters and 2 ethers were detected.

The analysis of ROAVs was conducted for the samples, as shown in Table 5, a total of 13 volatile flavor compounds with ROAVs >1 were identified as compounds significantly contributing to chicken meat flavor, including hexanal, heptanal, nonanal, 2-octenal, octanal, (*E,E*)-2,4-nonadienal, (*E,Z*)-2,4-decadienal, decanal, 1-hexanol, 1-octen-3-ol, 1-heptanol, 2-pentyl-furan, and dimethyl disulfide. Aldehydes dominate among the odor-active volatile compounds of stewed chicken due to their lower odor thresholds. Among these, hexanal (ROAV at 69.35–215.28), nonanal (ROAV at 58.61–95.24), heptanal (ROAV at 4.80–12.45), (*E,E*)-2,4-nonadienal (ROAV at 23.69–64.33), and (*E,Z*)-2,4-decadienal (ROAV at 108.32–173.48) imparted a grassy and fatty flavor, which may have contributed to the overall flavor of JC. 1-Octen-3-ol, known for its mushroom aroma, exhibited ROAV values (45.99–216.54) significantly higher than those of the other two alcohol compounds. 2-Pentyl-furan imparted a beany flavor to the chicken, while dimethyl disulfide, characterized by an onion aroma with an ROAV of 8.16, contributed exclusively to the flavor profile of LC. It is noteworthy that most of these above odor-active volatile compounds came from fat oxidation reactions. Possibly due to the relatively low temperature during stewing (Almela et al., 2010), the Maillard reactions and thiamine degradation hardly occur (Kosowska et al., 2017).

**Table 5:** Odor-active compounds (ROAVs >1) in two muscles of different chicken samples after stewing.

Number	Volatile compound	Odor description^a^	Threshold (μg/kg)^b^	Breed^c^ (B) and Muscle (M)						Significance (*P* value)		
				Leg muscle			Breast muscle					
				YC	LC	JC	YC	LC	JC	B	M	B × M
1	Hexanal	Grass (S)	5	146.49^ABa^	87.16^Ba^	215.28^Aa^	88.46^Aa^	69.35^Aa^	74.63^Ab^	NS	**	NS
2	Heptanal	Fatty (M)	2.8	10.18^Aa^	9.38^Aa^	12.45^Aa^	9.04^Aa^	4.80^Aa^	6.22^Ab^	NS	**	NS
3	Nonanal	Grassy (W)	1.1	71.06^Aa^	68.28^Aa^	73.53^Aa^	95.34^Aa^	ND	58.61^Aa^	**	NS	*
4	(*E*)-2-Octenal	Fragrance (W)	3	5.49^Aa^	2.88^Aa^	3.26^Aa^	ND	ND	2.88^Aa^	NS	**	NS
5	Octanal	Citrus (W)	0.59	50.23^Aa^	59.28^Aa^	67.59^Aa^	58.05^Aa^	21.17^Bb^	48.36^Aa^	NS	*	NS
6	(*E,E*)-2,4-Nonadienal	Fatty (S)	0.1	23.69^Aa^	ND	ND	ND	ND	64.33^Aa^	**	NS	***
7	(*E,Z*)-2,4-Decadienal	Oily (W)	0.027	173.48^Aa^	ND	ND	ND	ND	108.32^Aa^	*	NS	***
8	Decanal	Fruity (M)	3	ND	ND	ND	ND	ND	2.14^Aa^	**	**	**
9	1-Hexanol	Grassy (M)	5.6	6.79^Ba^	82.05^Aa^	ND	ND	ND	37.42^Aa^	NS	NS	**
10	1-Octen-3-ol	Mushroom (S)	1.5	79.67^Ab^	45.99^Aa^	76.21^Aa^	216.94^Aa^	ND	111.55^Ba^	***	**	***
11	1-Heptanol	Musty, Bean (W)	5.4	0.77^Ba^	0.82^Ba^	2.61^Aa^	ND	ND	1.97^Aa^	***	*	NS
12	2-Pentyl-furan	Bean (M)	5.8	3.49^Aa^	3.31^Aa^	2.32^Ab^	3.76^Aa^	2.07^Ba^	4.29^Aa^	NS	NS	*
13	Dimethyl disulfide	Onion (W)	1.1	ND	ND	ND	ND	8.16^Aa^	ND	**	*	**

“ND” stands for “Not Available “(The volatile compound was not detected in this sample, so the relative odor activity value cannot be calculated).

Different lowercase letters (a-b) indicate a significant difference (*P < 0.05*, differences between muscle) within the same breed. Different capital letters (A-B) indicate a significant difference (*P < 0.05*, differences between breed) within the same muscle type. Significance: *** *P < 0.001*, ** *P < 0.01*, * *P < 0.05*; NS, not significant.

a Odor description is the result obtained by sensory evaluators through olfactory evaluation. The perceived aroma intensity was categorized into three levels: Strong (S), Medium (M), and Weak (W).

b The thresholds of Odor-active compounds in water were obtained from a book titled Compilations of Odor Threshold Values in Air, Water and Other Media (Edition 2011).

c YC、LC and JC refers to Yellow feather chicken, Lohmann powder chicken and Jianmen native chicken, respectively.

#### 3.3.3 GC-IMS results of two muscles in different chicken samples after stewing

**Fig. 7:**
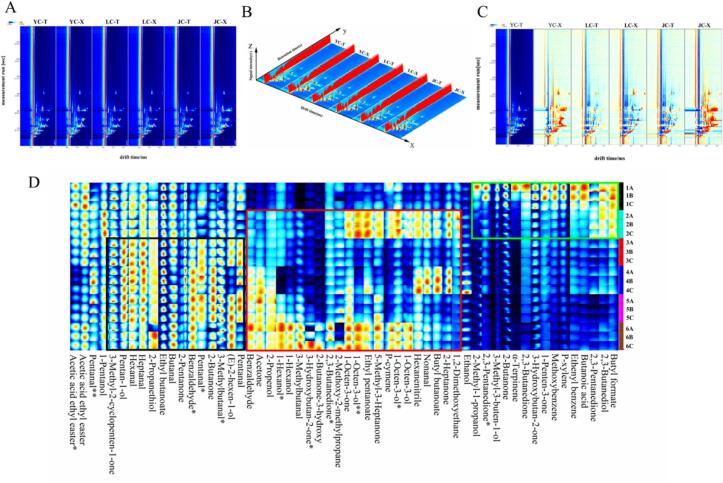
Volatile compounds of two muscles in different chicken samples after stewing. (A) Topographic plot of GC–IMS spectra; (B) 3D-topographic; (C) comparison results under the spectral diagram (YC-T) of one sample was selected as the reference. (D) Fingerprints of volatile compounds. 1A/1B/1C: leg muscle of Yellow feather chicken (YC-T); 2A/2B/2C: breast muscle of Yellow feather chicken (YC-X); 3A/3B/3C: leg muscle of Lohmann powder chicken (LC-T); 4A/4B/4C: breast muscle of Lohmann powder chicken (LC-X); 5A/5B/5C: leg muscle of Jianmen native chicken (JC-T); 6A/6B/6C: breast muscle of Jianmen native chicken (JC-X).

To further comprehensively investigate the differences in volatile compounds among different chicken breeds after stewing, the GC-IMS method was employed to identify these compounds, and the results were visualized. Fig. 7A illustrates the topographic map obtained from the GC-IMS analysis, with the vertical coordinate representing the retention time of the gas chromatogram and the horizontal coordinate representing the ion migration time. The overall background of this topographic map is blue, and each colored point represents a volatile flavor substance. The color shade of the point reflects the concentration of the substance: the closer the color is to red, the higher the concentration; the closer the color is to white, the lower the concentration. The figure shows that the retention time of most volatiles in chicken meat after stewing was 100–400 s, and the migration time was 1–8 ms. Fig. 7B shows the three-dimensional spectra of volatile compounds in the muscles of YC, LC, and JC after stewing, with the X, Y, and Z axes representing the migration time, retention time, and signal intensity of the ionic compounds, respectively. The graph clearly shows the similarities and differences of the volatile compounds in the chicken samples after stewing. In order to compare their differences more visually, Fig. 7C was analyzed using the leg muscle of YC as a reference. In the spectrogram, red color indicates that the concentration of volatile compounds in the samples is higher than the control, while blue color indicates that the concentration of compounds is lower than the control. The concentration of flavor substances was higher in LC and JC compared to those in the leg muscle of YC. Overall, the total amount and content of flavor substances were higher in LC and JC than in YC, which may be attributed to the occurrence of lipid degradation during the stewing process (Xu et al., 2024).

GC-IMS was used to qualitatively analyze the volatiles of different breeds of chicken meat after stewing as a whole. As shown in Table S3, a total of 42 volatile compounds were identified, including aldehydes, alcohols, ketones, esters, sulfides, aromatics and other compounds. Of these, some volatiles contained dimers and trimers produced by single compounds, probably due to the high concentration of volatile compounds, which produced multiple signals, which is consistent with the findings of Yao et al. (2022).

Fig. 7D demonstrates the fingerprints of volatile compounds in the leg and breast muscles of YC, LC, and JC after stewing. Throughout the fingerprint spectrum, the data were arranged in the form of sample numbers, e.g., 1 A/1B/1C represented three samples of YC leg muscle. In the area marked by the green rectangle, YC shows higher levels of 3-methyl-3-buten-1-ol, 2-methyl-1-propanol, 2,3-butanediol, 2,3-pentanedione, 2,3-butanedione, 1-penten-3-one, acetoin, 2-butanone, butyl formate, butanoic acid, and α-terpineol in leg muscle compared to LC and JC. The breast muscle from YC also shows relatively higher levels of butyl formate, 2,3-butanediol, and 2,3-pentanedione. In the area marked by the red rectangle, nonanal, 2-heptanone, and butyl butanoate were found at significantly higher levels in the breast muscle of YC and LC compared to JC, while 1-octen-3-ol, 1-octen-3-one, 5-methyl-3-heptanone, p-cymene, and ethyl pentanoate were more concentrated in the breast muscle of YC and JC. on the other hand, 3-methylbutanal, benzaldehyde, 1-hexanol, 2-propenol, and 2,3-butanedione, acetoin, acetone were present in the breast muscle of JC and LC, which conferred more nutty and sweet aroma to JC and LC chicken meat. In the area marked by black rectangles, hexanal, heptanal, 3-methylbutanal, pentanal, benzaldehyde, (*E*)-2-hexen-1-ol, 2-propanethiol, pentanol, and 2-butanone were found at significantly higher levels in LC and JC compared to YC. These volatile compounds, especially the aldehydes and alcohols, are closely related to the umami and nutty flavors of the chicken, likely making LC and JC stand out in these flavor characteristics. In summary, compared to YC, most volatile compounds exhibited higher concentrations in LC and JC. Furthermore, the results of GC-IMS were consistent with those of GC-O-MS.

### 3.4 Correlation analysis

**Fig. 8:**
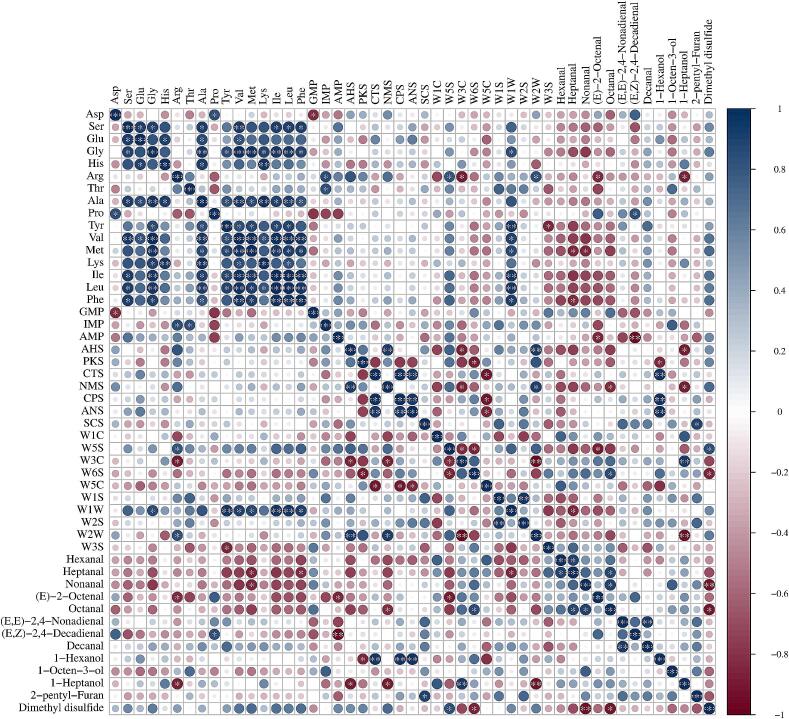
Correlation analysis between free amino acids, nucleotides, e-nose, e-tongue and key aroma compounds (ROAVs >1) of different chicken samples. The asterisks in the graph indicate the significance of the *r*-value (significance level is 0.05). Significance: *** *P < 0.001*, ** *P < 0.01*, * *P < 0.05.*

The Pearson method was used to analyze the correlation between free amino acids, nucleotides, electronic tongue, electronic nose, and key aroma compounds (ROAVs >1) in different chicken samples (|*r*| *> 0.8* and *p < 0.05*). As shown in Fig. 8, blue represents positive correlation and red represents negative correlation, and the larger absolute value of the correlation coefficient indicates a stronger correlation. The results show a significant positive correlation between umami amino acids Asp and Glu and sweet amino acids Pro, Ala, and Ser. IMP is positively correlated with Thr and Arg, while AMP is negatively correlated with (*E*)-2-octenal and (*E*,*Z*)-2,4-decadienal. In addition, bitter amino acids Tyr, Met, and Phe are significantly positively correlated with W1W responses, while negatively correlated with nonanal and hexanal. Further analysis revealed that the response values of CTS and ANS were significantly positively correlated with 1-hexanol, SCS with 2-pentyl-furan, and W5S with dimethyl disulfide, while ANS, NMS, and W2W were significantly negatively correlated with 1-heptanol. These results indicate a close relationship between volatile compounds and taste compounds.

## 4 Conclusions

The present study revealed significant differences in the flavor characteristics of different specialty chicken meat after stewing. Compared to the control group, both LC and JC exhibited superior flavor profiles after stewing, with LC particularly standing out for its umami. The combination of GC-O-MS and GC-IMS techniques was used to achieve a comprehensive profile of volatile flavor compounds in chicken after stewing. Among them, a total of 42 volatile compounds were detected using GC-IMS, including aldehydes, alcohols, ketones, esters, sulfides, and aromatics. Moreover, the content of volatile flavor compounds was higher in LC and JC than in YC. GC-O-MS, on other hand, detected 41 volatile compounds, and further analysis of the ROAV revealed that a total of 13 key flavor compounds had ROAVs >1, including hexanal, heptanal, nonanal, 2-octenal, octanal, (*E,E*)-2,4-nonadienal, (*E,Z*) -2,4-decadienal, decanal, 1-hexanol, 1-octen-3-ol, 1-heptanol, 2-pentyl-furan, dimethyl disulfide. Of these, the dominant saturated aldehydes and furan compounds were found in the highest amounts in JC, while dimethyl disulfide was only detected in the breast muscle of LC. In terms of taste compounds, LC had significantly higher (*P < 0.05*) contents of umami amino acids, bitter amino acids and AMP than the other breeds, whereas JC had a higher content of sweet amino acids. Combined with e-tongue analysis, it was found that the leg muscle of both LC and JC excelled in umami and sweetness scores. Additionally, LC exhibited the lowest cooking loss and highest shear characteristics, while JC showed the lowest protein and fat content. The information provided by the results of the study can be used to understand the components and differences in the flavors of specialty chicken meat, and provide a scientific basis for quality improvement of chicken meat from different breeds.

## CRediT authorship contribution statement

**Wenqian Lei:** Writing – original draft, Methodology, Investigation. **Zhaowei Cui:** Writing – original draft, Methodology, Investigation. **Wanqi Hu:** Writing – review & editing, Methodology. **Mengxuan Wang:** Writing – review & editing, Conceptualization. **Xuhua Chen:** Writing – review & editing, Methodology, Conceptualization. **Xiaojia Hu:** Writing – review & editing. **Prince Chisoro:** Writing – review & editing. **Dong Han:** Writing – review & editing, Conceptualization. **Jianchuan Zhou:** Writing – review & editing, Resources, Funding acquisition. **Chunhui Zhang:** Writing – review & editing, Resources, Funding acquisition.

## Declaration of competing interest

The authors declare that they have no known competing financial interests or personal relationships that could have appeared to influence the work reported in this paper.

## Data Availability

Data will be made available on request.

## Appendix A Supplementary data

Supplementary material Image 1
