# Supplementary material for: Characterization and comparison of flavor compounds in different specialty chicken meat after stewing
Source: Food Chem X. 2025 May 27;28:102589. doi: 10.1016/j.fochx.2025.102589 (PMC12167448; doi:10.1016/j.fochx.2025.102589)
Supplement: Supplementary file 1 — Supplementary material [file mmc1.docx]

**Caption**

Table S1. The basic components of two muscles in different chicken breeds.

Table S2. Description of the sensor performance of the PEN 3 electronic nose.

Table S3. GC-IMS qualitative analysis of volatile flavor compounds in two muscles of different chicken samples after stewing.

Table S4. Identification of volatile compounds in two muscles of different chicken samples after stewing by GC-MS.

Table S1. The basic components of two muscles in different chicken breeds.

| Index | Breed (B) and Muscle (M) | | | | | | | | Significance (*P* value) | | |
| --- | --- | --- | --- | --- | --- | --- | --- | --- | --- | --- | --- |
|  | Leg muscle | | |  | Breast muscle | | |  |  |  |  |
|  | YC | LC | JC | | YC | LC | JC | | B | M | B×M |
| Moisture (g/100g） | 73.313 ± 0.626^Aa^ | 73.550 ± 0.429^Aa^ | 71.866 ± 1.201^Ba^ | | 68.363 ± 0.315^Bb^ | 68.890 ± 1.230^Bb^ | 70.763 ± 0.375^Aa^ | | NS | *** | ** |
| Protein (g/100g） | 23.337 ± 1.191^Aa^ | 26.399 ± 2.517^Aa^ | 22.846 ± 0.866^Aa^ | | 23.148 ± 2.957^Aa^ | 25.058 ± 0.49^Aa^ | 24.879 ± 2.258^Aa^ | | NS | NS | NS |
| Fat (g/100g) | 1.93 ± 0.115^Aa^ | 1.70 ± 0.600^Aa^ | 1.47 ± 0.568^Aa^ | | 0.50 ± 0.200^Ab^ | 0.23 ± 0.057^Ab^ | 0.10 ± 0.001^Ab^ | | NS | *** | NS |

Note: Results were expressed as mean ± standard derivation. YC, LC and JC refers to Yellow feather chicken, Lohmann powder chicken and Jianmen native chicken, respectively. Different lowercase letters (a-b) indicate a significant difference (*P < 0.05*, differences between muscle) within the same breed. Different capital letters (A-B) indicate a significant difference (*P < 0.05*, differences between breed) within the same muscle type. Significance: *** *P < 0.001*, ** *P < 0.01*; NS, not significant.

Table S2. Description of the sensor performance of the PEN 3 electronic nose.

| Number | Sensor name | Performance description |
| --- | --- | --- |
| 1 | W1C | Benzene and aromatic compounds |
| 2 | W5S | Very sensitive to nitrogen oxides |
| 3 | W3C | Sensitive to aromatic compounds |
| 4 | W6S | Mainly hydrogen |
| 5 | W5C | Alkane, aromatic compounds |
| 6 | W1S | Sensitive to hydrocarbons |
| 7 | W1W | Sensitive to many sulfur organic compounds and terpenes |
| 8 | W2S | Alcohol, sensitive to aromatic compounds |
| 9 | W2W | Aromatic compounds and sulfur organic compounds |
| 10 | W3S | Sensitive to long chain alkanes |

Table S3. GC-IMS qualitative analysis of volatile flavor compounds in two muscles of different chicken samples after stewing.

| Count | Compound | CAS# | Formula | MW | RI | Rt [s] | Dt [ms] |
| --- | --- | --- | --- | --- | --- | --- | --- |
|  | 3-Methyl-2-cyclopenten-1-one | C2758181 | C_6_H_8_O | 96.1 | 983.9 | 415.224 | 1.42064 |
|  | 1-Octen-3-ol | C3391864 | C_8_H_16_O | 128.2 | 983 | 414.105 | 1.15865 |
|  | 1-Octen-3-ol* | C3391864 | C_8_H_16_O | 128.2 | 977.9 | 407.626 | 1.59868 |
|  | 1-Octen-3-ol** | C3391864 | C_8_H_16_O | 128.2 | 977.8 | 407.532 | 1.71136 |
|  | 1-Octen-3-one | C4312996 | C_8_H_14_O | 126.2 | 981.1 | 411.715 | 1.25296 |
|  | 2-Heptanone | C110430 | C_7_H_14_O | 114.2 | 883.7 | 306.549 | 1.69063 |
|  | (*E*)-2-Hexen-1-ol | C928950 | C_6_H_12_O | 100.2 | 865 | 290.18 | 1.32504 |
|  | 1-Hexanol | C111273 | C_6_H_14_O | 102.2 | 862.8 | 288.265 | 1.63796 |
|  | 1-Hexanol* | C111273 | C_6_H_14_O | 102.2 | 871.6 | 295.813 | 1.62415 |
|  | Butyl butanoate | C109217 | C_8_H_16_O_2_ | 144.2 | 994.9 | 429.261 | 1.82068 |
|  | Benzaldehyde | C100527 | C_7_H_6_O | 106.1 | 960.4 | 386.558 | 1.14905 |
|  | Benzaldehyde* | C100527 | C_7_H_6_O | 106.1 | 959.1 | 385.123 | 1.46907 |
|  | Hexanal | C66251 | C_6_H_12_O | 100.2 | 800.2 | 239.927 | 1.26167 |
|  | Butanoic acid | C107926 | C_4_H_8_O_2_ | 88.1 | 800.2 | 239.947 | 1.36487 |
|  | Ethyl butanoate | C105544 | C_6_H_12_O_2_ | 116.2 | 788.2 | 231.662 | 1.55754 |
|  | 1-Pentanol | C71410 | C_5_H_12_O | 88.1 | 768.1 | 218.157 | 1.25393 |
|  | 1-Pentanol* | C71410 | C_5_H_12_O | 88.1 | 762.2 | 214.366 | 1.51232 |
|  | 3-Methyl-3-buten-1-ol | C763326 | C_5_H_10_O | 86.1 | 728.3 | 193.753 | 1.15703 |
|  | Butyl formate | C592847 | C_5_H_10_O_2_ | 102.1 | 730.2 | 194.851 | 1.20427 |
|  | 2,3-Pentanedione | C600146 | C_5_H_8_O_2_ | 100.1 | 702.2 | 179.175 | 1.29741 |
|  | 2,3-Pentanedione* | C600146 | C_5_H_8_O_2_ | 100.1 | 713.6 | 185.389 | 1.29262 |
|  | 2-Pentanone | C107879 | C_5_H_10_O | 86.1 | 691.9 | 173.767 | 1.40266 |
|  | 1,2-Dimethoxyethane | C110714 | C_4_H_10_O_2_ | 90.1 | 651.3 | 157.622 | 1.28331 |
|  | Pentanal | C110623 | C_5_H_10_O | 86.1 | 699 | 177.502 | 1.19596 |
|  | Pentanal* | C110623 | C_5_H_10_O | 86.1 | 698 | 176.954 | 1.19586 |
|  | Pentanal** | C110623 | C_5_H_10_O | 86.1 | 680.2 | 168.708 | 1.18939 |
|  | 2-Butanone | C78933 | C_4_H_8_O | 72.1 | 633.1 | 151.026 | 1.05475 |
|  | 2-Butanone* | C78933 | C_4_H_8_O | 72.1 | 590.5 | 136.671 | 1.24251 |
|  | Ethyl acetate | C141786 | C_4_H_8_O_2_ | 88.1 | 614.6 | 144.606 | 1.09773 |
|  | Ethyl acetate* | C141786 | C_4_H_8_O_2_ | 88.1 | 611 | 143.412 | 1.33284 |
|  | 2-Methyl-1-propanol | C78831 | C_4_H_10_O | 74.1 | 630.2 | 150.016 | 1.15605 |
|  | 2,3-Butanedione | C431038 | C_4_H_6_O_2_ | 86.1 | 600.1 | 139.773 | 1.18572 |
|  | 2,3-Butanedione* | C431038 | C_4_H_6_O_2_ | 86.1 | 591.9 | 137.117 | 1.15446 |
|  | Butanal | C123728 | C_4_H_8_O | 72.1 | 602.9 | 140.71 | 1.28753 |
|  | Acetone | C67641 | C_3_H_6_O | 58.1 | 484.4 | 106.551 | 1.1207 |
|  | Ethanol | C64175 | C_2_H_6_O | 46.1 | 463.4 | 101.435 | 1.04894 |
|  | 2-Methoxy-2-methylpropane | C1634044 | C_5_H_12_O | 88.1 | 567.6 | 129.523 | 1.11886 |
|  | 3-Methylbutanal | C590863 | C_5_H_10_O | 86.1 | 657.7 | 160.006 | 1.16669 |
|  | 3-Methylbutanal* | C590863 | C_5_H_10_O | 86.1 | 657 | 159.738 | 1.39809 |
|  | 1-Penten-3-one | C1629589 | C_5_H_8_O | 84.1 | 681.1 | 169.071 | 1.29835 |
|  | Anisole | C100663 | C_7_H_8_O | 108.1 | 930.7 | 353.279 | 1.04985 |
|  | p-xylene | C106423 | C_8_H_10_ | 106.2 | 847.2 | 275.386 | 1.04985 |
|  | Ethenyl benzene | C100425 | C_8_H_8_ | 104.2 | 884.7 | 307.435 | 1.43488 |
|  | Hexanenitrile | C628739 | C_6_H_11_N | 97.2 | 872.6 | 296.706 | 1.25943 |
|  | Nonanal | C124196 | C_9_H_18_O | 142.2 | 1096.6 | 607.38 | 1.48119 |
|  | p-cymene | C99876 | C_10_H_14_ | 134.2 | 1020.2 | 467.776 | 1.15244 |
|  | 2-Propanol | C67630 | C_3_H_8_O | 60.1 | 494.3 | 109.055 | 1.09625 |
|  | Acetoin | C513860 | C_4_H_8_O_2_ | 88.1 | 744.6 | 203.38 | 1.05472 |
|  | Acetoin* | C513860 | C_4_H_8_O_2_ | 88.1 | 722.4 | 190.318 | 1.32316 |
|  | Acetoin** | C513860 | C_4_H_8_O_2_ | 88.1 | 729.2 | 194.241 | 1.32593 |
|  | 5-Methyl-3-heptanone | C541855 | C_8_H_16_O | 128.2 | 944.5 | 368.391 | 1.25554 |
|  | Ethyl pentanoate | C539822 | C_7_H_14_O_2_ | 130.2 | 901.2 | 323.003 | 1.26195 |
|  | Heptanal | C111717 | C_7_H_14_O | 114.2 | 885.1 | 307.789 | 1.3296 |
|  | 2,3-Butanediol | C513859 | C_4_H_10_O_2_ | 90.1 | 817.6 | 252.475 | 1.36529 |
|  | 2-Propanethiol | C75332 | C_3_H_8_S | 76.2 | 577.2 | 132.463 | 1.15478 |

Rt: Represents the retention time in the capillary GC column.

RI: Represents the retention index calculated using n-ketones C4–C9 as external standard on column.

Dt: Represents the drift time in the drift tube.

* Dimers formed in the IMS drift tube were represented by symbol “*”.

**Trimers formed in the IMS drift tube were represented by symbol “**”.

Table S4. Identification of volatile compounds in two muscles of different chicken samples after stewing by GC-MS.

| Number | volatile compound | Formula | CAS | RI^a^ | RI^b^ | Identification approach | Breed^c^ and Muscle | | | | | | |
| --- | --- | --- | --- | --- | --- | --- | --- | --- | --- | --- | --- | --- | --- |
|  |  |  |  |  |  |  | Leg muscle | | |  | Breast muscle | | |
|  |  |  |  |  |  |  | YC | LC | JC | | YC | LC | JC |
|  | Aldehydes (10) |  |  |  |  |  |  |  |  | |  |  |  |
| 1 | Hexanal | C_6_H_12_O | 66-25-1 | 1089 | 1110 | MS/RI | **+** | **+** | **+** | | **+** | **+** | **+** |
| 2 | Heptanal | C_7_H_14_O | 111-71-7 | 1195 | 1183 | MS/RI | **+** | **+** | **+** | | **+** | **+** | **+** |
| 3 | Nonanal | C_9_H_18_O | 124-19-6 | 1406 | 1387 | MS/RI | **+** | **+** | **+** | | **+** | **-** | **+** |
| 4 | (*E*)-2-Octenal | C_8_H_14_O | 2548-87-0 | 1440 | 1456 | MS/RI | **+** | **+** | **+** | | **-** | **-** | **+** |
| 5 | Benzaldehyde | C_7_H_6_O | 100-52-7 | 1532 | 1530 | MS/RI | **-** | **+** | **-** | | **-** | **-** | **-** |
| 6 | Octanal | C_8_H_16_O | 124-13-0 | 1301 | 1277 | MS/RI | **+** | **+** | **+** | | **+** | **+** | **+** |
| 7 | (*E,E*)-2,4-Nonadienal | C_9_H_14_O | 5910-87-2 | 1711 | 1702 | MS/RI | **+** | **-** | **-** | | **-** | **-** | **+** |
| 8 | (*E,Z*)-2,4-Decadienal | C_10_H_16_O | 25152-83-4 | 1777 | 1767 | MS/RI | **+** | **-** | **-** | | **-** | **-** | **+** |
| 9 | Glutaraldehyde | C_5_H_8_O_2_ | 111-30-8 | 1627 |  | MS | **-** | **-** | **+** | | **-** | **-** | **+** |
| 10 | Decanal | C_10_H_20_O | 112-31-2 | 1512 | 1481 | MS/RI | **-** | **-** | **-** | | **-** | **-** | **+** |
|  | Ketones (5) |  |  |  |  |  |  |  |  | |  |  |  |
| 11 | 2-Heptanone | C_7_H_14_O | 110-43-0 | 1192 | 1190 | MS/RI | **+** | **+** | **+** | | **+** | **+** | **+** |
| 12 | 2-Methyl-3-octanone | C_9_H_18_O | 923-28-4 | 1335 | 1323 | MS/RI | **+** | **+** | **-** | | **-** | **-** | **-** |
| 13 | 2,3-Octanedione | C_8_H_14_O_2_ | 585-25-1 | 1343 | 1325 | MS/RI | **+** | **+** | **-** | | **-** | **-** | **-** |
| 14 | 4-Hydrazinylpyridin-2(*1H*)-one | C_5_H_7_N_3_O | 106689-41-2 | 1327 |  | MS | **-** | **-** | **+** | | **-** | **-** | **-** |
| 15 | (*E,E*)**-**3,5-Octadien-2-one | C_8_H_14_O | 30086-02-3 | 1581 | 1570 | MS | **+** | **-** | **-** | | **-** | **-** | **-** |
|  | Alcohols (7) |  |  |  |  |  |  |  |  | |  |  |  |
| 16 | 1-Pentanol | C_5_H_12_O | 71-41-0 | 1266 | 1256 | MS/RI | **+** | **+** | **+** | | **+** | **+** | **+** |
| 17 | 1-Hexanol | C_6_H_14_O | 111-27-3 | 1367 | 1384 | MS/RI | **+** | **+** | **-** | | **-** | **-** | **+** |
| 18 | 1-Octen-3-ol | C_8_H_16_O | 3391-86-4 | 1466 | 1462 | MS/RI | **+** | **+** | **+** | | **+** | **-** | **+** |
| 19 | 1-Heptanol | C_7_H_16_O | 111-70-6 | 1472 | 1462 | MS/RI | **+** | **+** | **+** | | **-** | **-** | **+** |
| 20 | 1-Octanol | C_8_H_18_O | 111-87-5 | 1576 | 1559 | MS/RI | **-** | **+** | **-** | | **-** | **-** | **-** |
| 21 | (*Z*)-2-Octen-1-ol | C_8_H_16_O | 26001-58-1 | 1630 | 1552 | MS/RI | **+** | **+** | **+** | | **-** | **-** | **-** |
| 22 | 1-Hexacosanol | C_26_H_54_O | 506-52-5 | 1824 |  | MS | **-** | **-** | **-** | | **+** | **+** | **-** |
|  | Acids (2) |  |  |  |  |  |  |  |  | |  |  |  |
| 23 | Fumaramic acid | C_4_H_5_NO_3_ | 2987-87-3 | 1457 |  | MS | **+** | **+** | **-** | | **+** | **-** | **-** |
| 24 | Nonahexacontanoic acid | C_69_H_138_O_2_ | 40710-32-5 | 1772 |  | MS | **-** | **+** | **-** | | **+** | **+** | **-** |
|  | Esters (4) |  |  |  |  |  |  |  |  | |  |  |  |
| 25 | Ethyl oxamate | C_4_H_7_NO_3_ | 617-36-7 | 1875 |  | MS | **-** | **+** | **-** | | **-** | **-** | **-** |
| 26 | Triacontyl acetate | C_32_H_64_O_2_ | 41755-58-2 | 1844 |  | MS | **-** | **-** | **-** | | **+** | **-** | **-** |
| 27 | Octyl formate | C_9_H_18_O_2_ | 112-32-3 | 1576 | 1560 | MS/RI | **+** | **-** | **-** | | **-** | **-** | **+** |
| 28 | Hexyl formate | C_7_H_14_O_2_ | 629-33-4 | 1367 | 1382 | MS/RI | **-** | **-** | **+** | | **-** | **-** | **-** |
|  | Ethers (2) |  |  |  |  |  |  |  |  | |  |  |  |
| 29 | Propyl triacontyl ether | C_33_H_68_O | 1000406-28-6 | 1577 |  | MS | **-** | **-** | **-** | | **-** | **+** | **-** |
| 30 | Isobutyl tetradecyl ether | C_18_H_38_O | 1000406-32-7 | 1641 |  | MS | **-** | **-** | **-** | | **+** | **-** | **-** |
|  | Heterocyclics (9) |  |  |  |  |  |  |  |  | |  |  |  |
| 31 | 2-Pentyl-furan | C_9_H_14_O | 3777-69-3 | 1244 | 1235 | MS/RI | **+** | **+** | **+** | | **+** | **+** | **+** |
| 32 | 2-n-Butyl furan | C_8_H_12_O | 4466-24-4 | 1142 | 1138 | MS/RI | **-** | **-** | **-** | | **-** | **-** | **+** |
| 33 | Acetoin | C_4_H_8_O_2_ | 513-86-0 | 1295 | 1280 | MS/RI | **-** | **-** | **-** | | **-** | **+** | **-** |
| 34 | Dimethyl disulfide | C_2_H_6_S_2_ | 624-92-0 | 1079 | 1078 | MS/RI | **-** | **-** | **-** | | **+** | **-** | **-** |
| 35 | 3-Butenamide | C_4_H_7_NO | 28446-58-4 | 1085 |  | MS | **-** | **-** | **-** | | **+** | **-** | **-** |
| 36 | Methylpent-4-enylamine | C_6_H_13_N | 5831-72-1 | 1585 |  | MS | **+** | **-** | **+** | | **+** | **+** | **+** |
| 37 | n-Hexylmethylamine | C_7_H_17_N | 35161-70-7 | 2154 |  | MS | **-** | **-** | **+** | | **-** | **-** | **-** |
| 38 | 3-Methyl-2-butanamine | C_5_H_13_N | 34701-33-2 | 1779 |  | MS | **+** | **-** | **+** | | **-** | **-** | **+** |
| 39 | Propanamide | C_3_H_7_NO | 79-05-0 | 1856 | 1807 | MS/RI | **+** | **-** | **+** | | **-** | **-** | **+** |
|  | Hydrocarbons (2) |  |  |  |  |  |  |  |  | |  |  |  |
| 40 | 9-Octadecyne | C_18_H_34_ | 35365-59-4 | 1844 |  |  | **-** | **-** | **-** | | **-** | **+** | **-** |
| 41 | 1-Decene | C_10_H_20_ | 872-05-9 | 1050 | 1047 | MS/RI | **+** | **-** | **-** | | **-** | **-** | **-** |

“+” indicates that the compound was detected; “-” means that the compound was not detected.

^a^ Retention index (RI) relative to n-alkanes in the polar column (DB-WAX)

^b^ RIs were obtained from the NIST Chemistry WebBook (<http://webbook.nist.gov/chemistry/>).

^c^ YC, LC and JC refers to Yellow feather chicken, Lohmann powder chicken and Jianmen native chicken, respectively.
